# Quantifying Neutralizing Antibodies in Patients with COVID-19 by a Two-Variable Generalized Additive Model

**DOI:** 10.1128/msphere.00883-21

**Published:** 2022-02-02

**Authors:** Kuan-Ting Liu, Yu-Nong Gong, Chung-Guei Huang, Peng-Nien Huang, Kar-Yee Yu, Hou-Chen Lee, Sun-Che Lee, Huan-Jung Chiang, Yu-An Kung, Yueh-Te Lin, Mei-Jen Hsiao, Po-Wei Huang, Sheng-Yu Huang, Hsin-Tai Wu, Chih-Ching Wu, Rei-Lin Kuo, Kuan-Fu Chen, Chuan-Tien Hung, Kasopefoluwa Y. Oguntuyo, Christian S. Stevens, Shreyas Kowdle, Hsin-Ping Chiu, Benhur Lee, Guang-Wu Chen, Shin-Ru Shih

**Affiliations:** a Research Center for Emerging Viral Infections, College of Medicine, Chang Gung University, Taoyuan, Taiwan; b Graduate Institute of Biomedical Science, College of Medicine, Chang Gung University, Taoyuan, Taiwan; c Department of Laboratory Medicine, Linkou Chang Gung Memorial Hospital, Taoyuan, Taiwan; d Department of Medical Biotechnology and Laboratory Science, College of Medicine, Chang Gung University, Taoyuan, Taiwan; e Division of Infectious Diseases, Department of Pediatrics, Linkou Chang Gung Memorial Hospital, Taoyuan, Taiwan; f Graduate Institute of Medical Biotechnology and Laboratory Science, College of Medicine, Chang Gung University, Taoyuan, Taiwan; g Department of Medicine, College of Medicine, Chang Gung University, Taoyuan, Taiwan; h Department of Otolaryngology/Head & Neck Surgery, Chang Gung Memorial Hospital, Linkou, Taoyuan, Taiwan; i Division of Asthma, Allergy, and Rheumatology, Department of Pediatrics, Linkou Chang Gung Memorial Hospital, Taoyuan, Taiwan; j Artificial Intelligence Research Center, Chang Gung University, Taoyuan, Taiwan; k Department of Computer Science and Information Engineering, School of Electrical and Computer Engineering, College of Engineering, Chang Gung University, Taoyuan, Taiwan; l Department of Emergency Medicine, Chang Gung Memorial Hospital, Keelung, Taiwan; m Clinical Informatics and Medical Statistics Research Center, Chang Gung University, Taoyuan, Taiwan; n Community Medicine Research Center, Chang Gung Memorial Hospital, Keelung, Taiwan; o Department of Microbiology, Icahn School of Medicine at Mount Sinai, New York, New York, USA; p Research Center for Chinese Herbal Medicine, Research Center for Food and Cosmetic Safety, Graduate Institute of Health Industry Technology, College of Human Ecology, Chang Gung University of Science and Technology, Taoyuan, Taiwan; University of Saskatchewan

**Keywords:** SARS-CoV-2, enzyme-linked immunosorbent assay, neutralizing antibody, receptor-binding domain, spike protein, two-variable generalized additive model

## Abstract

Considering the urgent demand for faster methods to quantify neutralizing antibody titers in patients with coronavirus (CoV) disease 2019 (COVID-19), developing an analytical model or method to replace the conventional virus neutralization test (NT) is essential. Moreover, a “COVID-19 immunity passport” is currently being proposed as a certification for people who travel internationally. Therefore, an enzyme-linked immunosorbent assay (ELISA) was designed to detect severe acute respiratory syndrome CoV 2 (SARS-CoV-2)-neutralizing antibodies in serum, which is based on the binding affinity of SARS-CoV-2 viral spike protein 1 (S1) and the viral spike protein receptor-binding domain (RBD) to antibodies. The RBD is considered the major binding region of neutralizing antibodies. Furthermore, S1 covers the RBD and several other regions, which are also important for neutralizing antibody binding. In this study, we assessed 144 clinical specimens, including those from patients with PCR-confirmed SARS-CoV-2 infections and healthy donors, using both the NT and ELISA. The ELISA results analyzed by spline regression and the two-variable generalized additive model precisely reflected the NT value, and the correlation between predicted and actual NT values was as high as 0.917. Therefore, our method serves as a surrogate to quantify neutralizing antibody titer. The analytic method and platform used in this study present a new perspective for serological testing of SARS-CoV-2 infection and have clinical potential to assess vaccine efficacy.

**IMPORTANCE** Herein, we present a new approach for serological testing for SARS-CoV-2 antibodies using innovative laboratory methods that demonstrate a combination of biology and mathematics. The traditional virus neutralization test is the gold standard method; however, it is time-consuming and poses a risk to medical personnel. Thus, there is a demand for methods that rapidly quantify neutralizing antibody titers in patients with COVID-19 or examine vaccine efficacy at a biosafety level 2 containment facility. Therefore, we used a two-variable generalized additive model to analyze the results of the enzyme-linked immunosorbent assay and found the method to serve as a surrogate to quantify neutralizing antibody titers. This methodology has potential for clinical use in assessing vaccine efficacy.

## INTRODUCTION

Severe acute respiratory syndrome coronavirus 2 (SARS-CoV-2) is an enveloped, positive-sense, and single-stranded RNA virus belonging to the family *Coronaviridae*. It is a type of the SARS-related coronavirus species (SARSr-CoV) that caused the SARS outbreak at the end of February 2003 (1). SARS-CoV-2 causes coronavirus disease 2019 (COVID-19), which has erupted into a global pandemic.

There is a need for a reliable assay to quantify neutralizing antibodies for assessing the herd immunity and humoral protective immunity in recovered patients and vaccine recipients (2). Presently, two traditional antibody tests are in use. The first is a virus neutralization test (NT) that detects neutralizing antibodies in the blood of patients. The NT requires the handling of live SARS-CoV-2 and a specialized biosafety level 3 (BSL3) containment facility. Furthermore, it is tedious and time-consuming (requires 3 to 5 days) to obtain the results. The second assay is a pseudovirus NT, which is similar to the NT that uses a live pseudovirus and requires a BSL2 facility (3, 4). Additionally, it takes 2 to 3 days to determine pseudovirus NT results. Furthermore, the available lateral flow rapid tests and IgG and IgM enzyme-linked immunosorbent assays (ELISA) can detect neutralizing antibodies; however, they cannot accurately quantify them (5). Although the receptor-binding domain (RBD) is the major binding region for neutralizing antibodies, other regions, such as the N-terminal domain (NTD) of viral spike protein 1 (S1), also bind to neutralizing antibodies (6). Regression studies on the linear relationship between SARS-CoV-2 S1 or RBD ELISA binding assays and NT titers show significant *P* values or *R*^2^ values in the interquartile range of the optical density (OD). However, large variations were found in the lower 25th or the higher 75th percentile (5, 7). This suggests that a simple linear regression model may not be sufficient to deliver the desired correlations between the OD values and NT titers. Thus, a dynamic predictor function is needed not only to model their nonlinear relationship but also to empirically decipher hidden patterns in the data.

We designed an assay that combined each of the S1 and RBD OD values and applied spline-based generalized additive model (GAM) regression analysis to predict NT titers. This method effectively increased the correlation between the live-virus NT assay and two ELISA-based binding assays using S1 and the RBD. This study provides a new perspective and an analytical model as an alternative to the traditional serological test to quantify neutralizing antibody titers in clinics.

## RESULTS

### SARS-CoV-2 structural proteins strongly bound to neutralizing antibodies.

**FIG 1:**
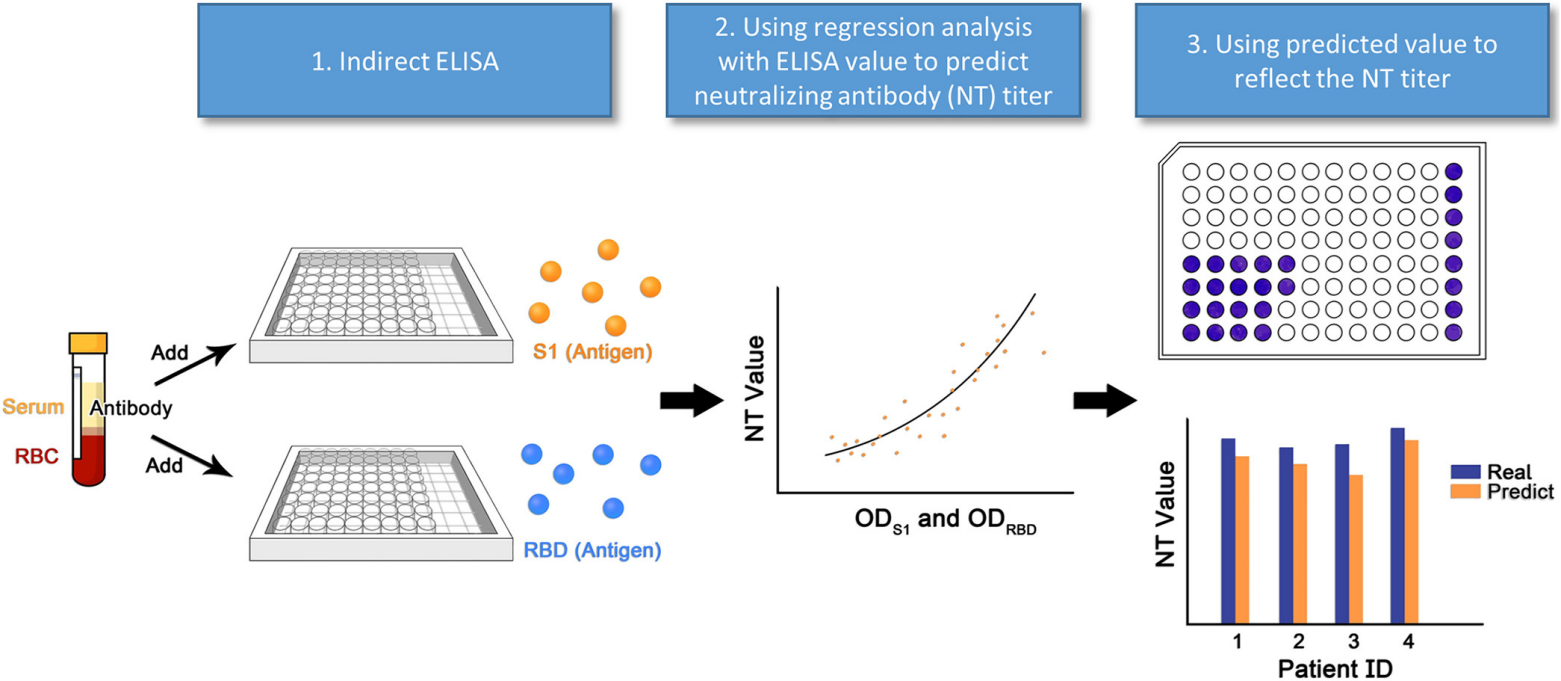
Schematic diagram of the ELISA binding assay and two-variable generalized additive analysis. Principles and methods for converting the ELISA binding affinity to SARS-CoV-2 neutralizing antibody (NT) titer are displayed. First, the indirect ELISA based on S1 and the RBD was established to detect antibody responses. The serum samples from patients with COVID-19 were used in both the ELISA and NT. Second, the ELISA results of S1 and the RBD and NT titers were used for training a spline-based generalized additive model. Eventually, the NT titers using the model were predicted, and the titers reflect the actual NT titer. RBC, red blood cells.

Figure 1 illustrates our strategy for the assay and data analysis. Our design was based on the combination of the RBD and S1 proteins, which are crucial binding regions for neutralizing antibodies. A two-variable GAM was applied to the two OD values of S1 and the RBD to predict NT titers, which outperformed simple linear regression.

**FIG 2:**
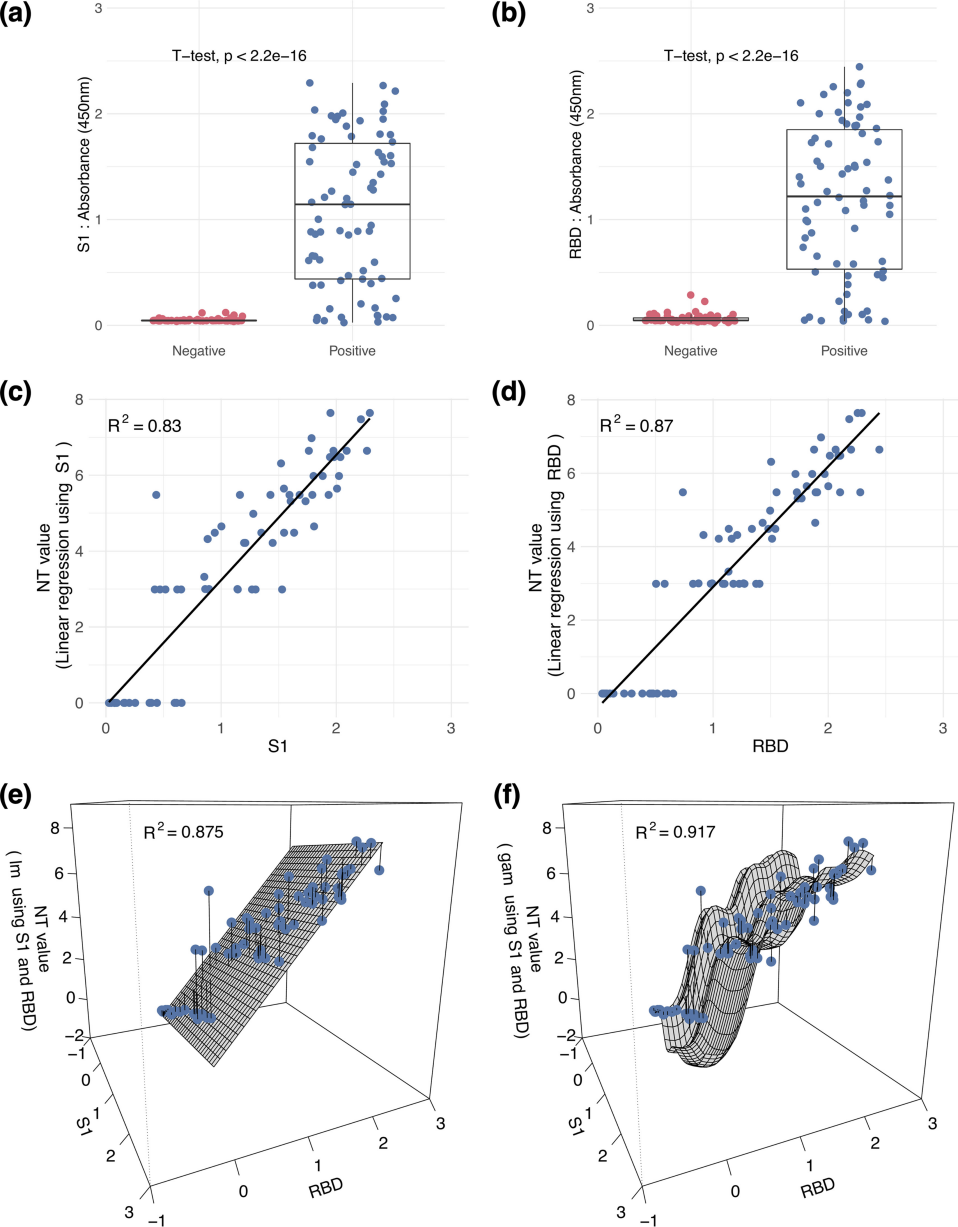
Detection and prediction of SARS-CoV-2-neutralizing antibody. (a to d) The binding ability of SARS-CoV-2 S1 and the RBD were measured in 74 samples from patients with COVID-19 and 70 negative controls. Linear regression was calculated for correlating the binding abilities of S1 (c) and the RBD (d) and two-variable regression versus the log_10_ neutralizing antibody titers of these 74 patients with COVID-19 (e). (f) Finally, the two-variable generalized additive method was applied. Correlation and linear regression analysis used Pearson’s correlation coefficients.

We detected the binding of the full S1 and RBD proteins in 74 patients with COVID-19 and compared them to that in 70 negative controls. Significant *P* values (Fig. 2a and b) were found separating the patient group from the control group. Furthermore, we analyzed the correlation between the neutralizing antibody titer and the S1 and RBD antibody responses, respectively. The binding of S1 (*R*^2^ = 0.830) and the RBD (*R*^2^ = 0.870) was well correlated with the log_10_-transformed actual NT neutralizing antibody titer (Fig. 2c and d).

### Spline-based two-variable GAM ELISA using S1 and the RBD.

Based on our findings that suggest that S1 and the RBD are superior predictors of the actual NT titer, we further applied a two-variable linear regression model, as well as a spline-based GAM, for correlating the binding capacity and actual NT titer. The two-variable linear regression model (Fig. 2e) had an *R*^2^ of 0.875, which is similar to that of the one-variable linear regression of the RBD in Fig. 2d. The GAM using S1 and the RBD as two variables has the highest *R*^2^ value, 0.917, between the binding capacity and actual NT titer (Fig. 2f). Additionally, L2 norms were 8.846, 7.687, 7.680, and 6.255 according to Fig. 2c to f, respectively.

To examine this GAM, a 5-fold cross-validation was applied, leading to high *R*^2^ values of 0.931, 0.914, 0.941, 0.920, and 0.954. The Akaike information criterion (AIC) was further used to compare the relative qualities for model selection. AIC values using models in Fig. 2c to f are 220.133, 199.356, 201.220, and 199.343, respectively. The two-variable GAM (Fig. 2f) shows the highest *R*^2^ value (0.917), as well as the lowest AIC value (199.343). All the above results demonstrated that the GAM using two variables outperformed the one- or two-variable linear regression models, which serves as a surrogate for the live-virus NT. Additionally, the same AIC value was obtained for this two-variable GAM with different smoothing parameters (as options of GCV.Cp, REML, and ML in the mgcv package).

### Comparison of correlation between assays to quantify the neutralizing antibody titer.

As pseudovirus is commonly considered a surrogate for a real virus to detect neutralizing antibodies, we verified if the pseudovirus NT or other binding assays reflect the actual NT titer. We used two different systems of the pseudotyped NT to quantify the neutralizing ability. The first one was the lentiviral pseudovirus NT, where the correlation between the lentiviral pseudovirus NT titer and SARS-CoV-2 NT titer (*R*^2^) was 0.385 (see Fig. S1a in the supplemental material). The second one used a vesicular stomatitis virus (VSV)-based pseudovirus, which is a pseudotyped SARS-CoV-2 viral particle (CoV2pp); this test used VSV bearing the *Renilla* luciferase gene instead of its G glycoprotein, and the correlation between the CoV2pp NT titer and SARS-CoV-2 NT titer was 0.427 (Fig. S1b). Furthermore, we performed an analysis using the SARS-CoV-2 surrogate virus NT kit from GenScript with the same serum samples and determined the correlation between the inhibition rate and NT titer; the *R*^2^ was 0.721 (Fig. S1c). The binding assays used in this study apparently showed a higher correlation with NT titer and were found to be safer and quicker serological assays.

10.1128/msphere.00883-21.1 FIG S1 Correlation between the pseudovirus neutralization test and surrogate neutralization test against authentic SARS-CoV-2 NT values. Analyses of linear correlations of SARS-CoV-2 NT values with the lenti-pseudovirus neutralization test (a) and VSV-pseudovirus neutralization test (b) were performed. The scales of these pseudovirus neutralization tests were log_10_ transformed. (c) Linear correlation was calculated between the SARS-CoV-2 NT value and inhibition rate using the GenScript SARS-CoV-2 surrogate virus neutralization test (sVNT). All of these correlations and linear regression analyses used Pearson’s correlation coefficients calculated with GraphPad Prism. Download FIG S1, TIF file, 0.5 MB. Copyright © 2022 Liu et al. 2022 Liu et al. https://creativecommons.org/licenses/by/4.0/ This content is distributed under the terms of the Creative Commons Attribution 4.0 International license.

## DISCUSSION

In this study, we developed a unique serological test platform that is safer and quicker than the traditional NT for quantifying neutralizing antibodies in serum. We detected the antibody responses of 74 positive serum samples from patients with COVID-19, which was confirmed via PCR, and 70 negative serum samples from non-COVID-19 donors. Based on our ELISA system, we developed a two-variable GAM regression model to incorporate the ELISA results for both S1 and the RBD; this model could accurately predict the NT titer with the conversion value from the optical density (OD) of S1 and the RBD. Overall, this approach serves as a surrogate NT to replace the conventional NT and can meet the urgent demand for a serological test.

In our strategy, the S1 and RBD proteins served as antigens to quantify the NT titer, although not all antibodies binding to these proteins are neutralizing antibodies. However, we considered that the antibodies binding to the S1 and RBD proteins have a greater probability of blocking virus entry (8–10). Furthermore, the RBD is considered a major binding region for neutralizing antibodies, and S1 covers the RBD and several other regions, which are also critical for neutralizing antibody binding (11). Thus, we used both S1 and the RBD protein to achieve the conversion and obtain a highly accurate NT titer. Additionally, titer values from the GAM spline regression model combining S1 and the RBD protein presented the best correlation (*R*^2^ = 0.917) (Fig. 2f) with the actual NT values.

The global COVID-19 outbreak started in December 2019 (12), and there are still no effective therapies to reduce morbidity and mortality. However, most patients who recovered from the infection produced neutralizing antibodies against SARS-CoV-2. A neutralizing antibody is a critical indicator of a previous infection. Thus, neutralizing antibody detection is a significant marker during a viral pandemic for controlling the disease. The conventional NT is the standard approach for quantifying neutralizing antibodies in serum, although it poses biosafety concerns and is time-consuming. Several vaccines that are based on the production of neutralizing antibodies in recipients have already entered clinical trials (13, 14), making antibody detection crucial (15). Therefore, a new and quick serological test is urgently needed to substitute for the conventional NT (16).

Surrogates or ELISA-based methods have been developed to estimate the authentic virus-neutralizing assays, based on the ability to block RBD–angiotensin-converting enzyme 2 (ACE2) interactions or antibody binding affinity to spike proteins (16–18). The advantage of an RBD-ACE2 surrogate approach is to provide a rapid and safe screening method for neutralizing antibody responses. However, the RBD-ACE2 surrogate is unable to represent the binding of neutralizing antibodies across the S1 region and vice versa. Instead, we incorporated both RBD and S1 ELISAs to increase the correlation with NT titers. Furthermore, the performance of the correlation with NT titers depends on statistical measurements and data sampling. Although there have been some studies showing antibody responses that are highly or moderately correlated (Spearman *r* > 0.6) with NT results, these correlations were calculated by dividing samples into different time points or by using ELISA endpoint titer groups (19, 20). Another study showed high correlations of NT values with a surrogate VNT (sVNT; Pearson *r*^2^ = 0.8591) or a pseudovirus-based VNT (pVNT; Pearson *r*^2^ = 0.7678); however, the Pearson correlation between NT values and indirect RBD ELISA titers was lower than 0.6 (Table S2 [16]), Compared to our ELISA-based experiments measuring NT titers, we showed a higher Pearson correlation of 0.87 (Fig. 2d). To conclude, surrogates or ELISA-based methods are ideal to mimic the authentic virus-neutralizing assays, but it would be biased to directly compare those correlations with NT predictions with different experimental systems and data sampling approaches or when using statistical measurements.

Two-variable GAM showed a high correlation (Pearson *r*^2^ > 0.9) between ELISA and NT results. It is expected that two independent variables, S1 and the RBD, representing more neutralizing antibody responses, are capable of better reflecting the accurate NT value. However, it would be biased to directly compare correlations using different ELISA-based systems which were based on different antigen expression sources, serum dilutions, and color reagent sensitivities. Moreover, the NT assay in this study was based on Vero E6 cells, which are commonly used to isolate and propagate SARS-CoV-2. Previous studies showed that Vero E6 cells lack TMPRSS2, a critical cellular serine protease required for virus entry into host cells. Vero E6/TMPRSS2 cells have been engineered to be more susceptible to SARS-CoV-2 or other coronavirus infections (4, 21, 22). Therefore, Vero E6/TMPRSS2 cells reflect an authentic infection more so than Vero E6 cells. Although some limitations in such surrogates remain, we provided new insights into serological testing to predict SARS-CoV-2 NT values by using an ELISA-based surrogate.

After SARS-CoV-2 infection, the host produces antibodies against several viral proteins, among which the N antibody is the most abundant (23). Therefore, many commercially available kits or rapid tests have been designed to detect the N antibody in serum (24). Additionally, the N protein is a conserved region in different strains. We speculated that cross-reactivity occurred in people who had been previously infected with other coronavirus strains, such as human CoV (HCoV)-OC43, HCoV-229E, and HCoV-NL63 (25). Therefore, S1 and the RBD may be more appropriate diagnostic markers in serological testing.

Presently, a critical issue is how long the neutralizing antibodies persist in infected people. Previous studies showed that the antibodies persisted in serum for 2 to 3 months after infection and then decreased slightly. The actual decrease was also dependent on the severity of the disease in the patients (26). Besides, it is still unknown how many neutralizing antibodies existing in the body will protect the host from getting SARS-CoV-2 infection or reinfection. However, previous studies demonstrated that neutralizing antibody levels are highly predictive of immune protection (27). There are many people getting SARS-CoV-2 infection or vaccinated with vaccines in the world. Therefore, it will be a significant issue to know the relationship between the neutralizing antibodies and their protection rate in the future. An immune passport based on the neutralizing antibody response has been proposed to allow people to return to work or travel, although some controversies remain regarding whether people with neutralizing antibodies have a lower chance of reinfection (28). Therefore, neutralizing antibody responses for these individuals must be traced for an extended period in the future. This information is critical for vaccination, and it can suggest how long the protection will persist.

In this study, we demonstrated a new diagnostic method that is safer and quicker for quantifying neutralizing antibodies. We found that the predicted values by two-variable GAM combining S1 and RBD antibody levels highly correlated with the neutralizing antibody titers. Although the conventional NT is an important method for confirming antibody responses to the live virus, our method can resolve the challenge of quantifying neutralizing antibodies for multiple samples in a short time.

## MATERIALS AND METHODS

### Panels of human sera used in this study.

COVID-19-positive and -negative panels were purchased from Access Biologicals (Vista, CA, USA); convalescent-phase serum samples from patients who had been diagnosed with COVID-19 were actively collected using the Roche swab (Basel, Switzerland). The patients were confirmed COVID-19-positive donors (ethics approval no. 2020-CR00207868). Clinical specimens from patients with COVID-19 or non-COVID-19 respiratory tract infections were collected from Chang Gung Memorial Hospital (ethics approval no. 202001951B0).

### Cell and virus samples.

Human embryonic kidney (HEK293T) cells (ATCC CRL-3216; ATCC, Manassas, VA, USA) and clone E6 (Vero-E6) cells (ATCC CRL-1586) were maintained in Dulbecco’s modified Eagle’s medium (DMEM) supplemented with 10% fetal bovine serum (FBS). SARS-CoV-2 was obtained from Chang Gung Memorial Hospital (CGUMH-CGU-04, GISAID accession no. EPI_ISL_415742) and used in the virus NT on Vero E6 cells (ATCC CRL-1586).

### ELISA.

For the indirect ELISA, a 96-well plate was coated with 2 μg/ml of the S1, RBD, and N proteins (Sino Biological, China) diluted in phosphate-buffered saline (PBS) overnight at 25°C. Each well was blocked with 300 μL of StartingBlock T20 blocking buffer (Thermo Fischer Scientific, Waltham, MA, USA) for 1 h at 25°C. Heat-inactivated convalescent-phase serum samples from COVID-19 patients and healthy donors were diluted in blocking buffer at a ratio of 1:200, and then 100 μL of the samples was added to the 96-well plate in duplicate. After the plates were washed, horseradish peroxidase (HRP)-tagged anti-human (IgG, IgM, and IgA) antibodies (Abcam, Cambridge, UK) were diluted 1:10,000 with blocking buffer and added to the wells (100 μL/well), and the plate was then incubated for 1 h at 25°C. Samples with N antibodies were incubated only for 30 min at 25°C because of the higher signal. The chromogenic reagent 3,3′,5,5′-tetramethylbenzidine (TMB) was mixed with an equal volume of color A and color B (R&D Systems, Minneapolis, MN, USA). The TMB reaction time for the S1 and RBD ELISA was 5 min, whereas that for the N protein ELISA was 10 min. After the reaction, stop solution (R&D Systems) was added to the wells and the OD was measured immediately at 450 nm using a Synergy 2 microplate reader (Bio-Tek, Winooski, VT, USA).

### Pseudotyped SARS-CoV-2 spike lentivirus.

The pseudotyped SARS-CoV-2-S Luc lentiviruses were prepared in 293T cells. The vectors used were pcDNA3.1, pCMVdeltaR8.91, and pLAS2w.FLuc.Ppuro to express the spike protein on the viral surface. After expression, the virus was selected with puromycin to quantify the titer (pseudotyped SARS-CoV-2-S Luc lentivirus was provided by the National RNAi Core Facility, Academia Sinica, Taiwan).

### Pseudovirus NT.

ACE2-transfected HEK293T cells were seeded (6 × 10^4^ cells/well) in a 96-well plate and incubated for 24 h at 37°C with 5% CO_2_. Pseudotyped SARS-CoV-2 spike lentivirus (6 × 10^3^ relative infection units) was preincubated with different dilutions of heat-inactivated convalescent-phase sera from patients with COVID-19 in duplicate for 1 h at 37°C. After incubation, we removed 50 μL of medium from each well and added 50 μL of the virus and serum mixture to the wells. Then, 24 h postinfection, the medium from the wells was removed, and 100 μL of 10% FBS in DMEM was added. Finally, 48 h postinfection, 50 μL medium was removed and 50 μL of the substrate of Bright-Glo (Promega, Madison, WI, USA) was added to lyse the cells. The luminescence signal was measured using a Synergy 2 microplate reader.

For the VSV-based pseudovirus neutralization assay, Vero-CCL81 cells were seeded at 2 × 10^4^ cells per well in a 96-well plate and incubated at 37°C for 24 h. Pseudotyped SARS-CoV-2 viral particle (CoV2pp) was provided by Benhur Lee from the Icahn School of Medicine, Mount Sinai, NY. The serum samples from all patients began to neutralize at a 10-fold dilution in DMEM. The samples were then serially diluted 4-fold in DMEM with 10% FBS. Vero-CCL81 cells were infected with diluted serum and CoV2pp in triplicate. The infected cells were washed with PBS and lysed with passive lysis buffer 20 h postinfection, and *Renilla* luciferase activity was measured using the *Renilla* luciferase assay kit (Promega).

### GenScript surrogate virus NT.

Positive-control and negative-control serum samples and serum samples diluted with HRP-RBD protein at a volume ratio of 1:1 were mixed in tubes and incubated at 37°C for 30 min in duplicate. The mixtures were added to the corresponding wells and incubated at 37°C for 15 min. The plate was washed four times with a wash buffer. Then, 100 μL of TMB solution was added to each well, and the plate was incubated in the dark at 25°C for 15 min. Stop solution (50 μL) was added to each well to stop the reaction. The OD was measured immediately at 450 nm using a Synergy 2 microplate reader. The inhibition rate was calculated as (1 − OD value of sample/OD value of negative control) × 100%.

### NT.

Vero E6 cells were consistently maintained in minimal essential medium (MEM) supplemented with 10% (vol/vol) FBS. SARS-CoV-2 was propagated in Vero E6 cells in a maintenance medium consisting of MEM supplemented with 2% FBS. For the NT test, Vero E6 cells were seeded (2.5 × 10^4^ cells/well) in a 96-well plate and incubated at 37°C with 5% CO_2_ for 18 h. After the incubation, the medium was replaced with 2% MEM for further analysis. Serum samples were inactivated at 56°C for 30 min before use and diluted in MEM at a ratio of 1:8 in quadruplicate. Twofold serially diluted serum samples were mixed with an equal volume of virus suspension at 100-fold the median tissue culture infectious dose (TCID_50_).The mixture was incubated for 1 h at 37°C, and then Vero E6 cells were incubated in 2% MEM for 5 days at 37°C. On postinfection day 5, the cells were fixed with 10% formaldehyde and stained with 0.1% crystal violet. Serum neutralization titers were analyzed using the Reed-Muench method to calculate the logarithm 50% endpoint.

### Generalized additive model.

The regression approach was applied to model the relationship between a dependent variable (NT value) and independent variables (or predictors, including two OD values at 450 nm for S1 and the RBD). Instead of using linear regression to model this relationship, we used spline regression to fit a smooth curve with a series of piecewise polynomials, and spline-based GAM was used to estimate multiple smooth relationships simultaneously (29–32). This spline-based GAM (using S1 and the RBD as two predictors) was applied to predict NT titer by adding up multiple smooth functions. Specifically, GAM could empirically capture the impact of variables through smooth spline functions, as follows:

(1) $$\text{g}\left[E\left(Y\right)\right]=\text{α}+S_{1}\left(x_{1}\right)+S_{2}(x_{2})\;\ldots +S_{p}(x_{p})$$

where *Y* is the dependent variable, *E*(*Y*) denotes the expected value, and g(*Y*) as a link function links the expected value to independent variables from *x_1_*, *x_2_*, …, *x_p_* and *S_1_*(*x_1_*), *S_2_*(*x_2_*), …, *S_p_*(*x_p_*), which denote smooth nonparametric functions. The GAM allowed the flexible estimation of the predictive patterns without *a priori* knowledge, providing a more efficient and less model-dependent way. In this model, the nonparametric smooth spline showed the shape of predictor functions empirically determined by the data, which outperformed the parametric function by a relatively small set of parameters.

All the regression modeling in this study was performed by the mgcv (version 1.8-35) package of the R programing language (version 3.6.1) (32, 33). This package offers GAM and smoothness estimation. In detailed settings of GAM function, we used the cubic regression (cr) splines for the smoothing basis (bs) argument, a smoothing parameter of 0.6 for the smoothing parameter (sp) argument, and GCV.Cp as the default smoothing parameter estimation method. An AIC (34) was applied to compare the relative qualities of models to avoid choosing an overfitting model. A lower AIC value indicates a balance between the model fitting and complexity. Fivefold cross-validation was also performed for evaluating models. All data and codes are publicly available at https://github.com/yngong/two_variable_gam.

To estimate the performance of linear regression and spline-based GAM approaches, the coefficient of determination (*R*^2^) and L2 norm were calculated using the following two equations:

(2) $$R^{2}=\;\frac{\sum_{i=1}^{n}{\left(y_{i}-\bar{y_{i}}\right)}^{2}-\sum_{i=1}^{n}{\left(y_{i}-{\hat{y}}_{i}\right)}^{2}}{\sum_{i=1}^{n}{\left(y_{i}-\bar{y_{i}}\right)}^{2}}$$

(3) $$\text{L2 norm}=\sqrt{\overset{n}{\underset{i=1}{\sum }}{\left(y_{i}-{\hat{y}}_{i}\right)}^{2}}$$

where, *n* is the number of samples and *y*, $\hat{y}$, and $\bar{y}$ are the real, predicted, and mean values, respectively. All statistical analyses were conducted and visualized using the ggplot2 package (version 3.3.3) (35).

### Data availability.

Code used in this study can be found at https://github.com/yngong/two_variable_gam.
