# Supplementary figures and images for: Quantifying Neutralizing Antibodies in Patients with COVID-19 by a Two-Variable Generalized Additive Model
Source: mSphere. 2022 Feb 2;7(1):e00883-21. doi: 10.1128/msphere.00883-21 (PMC8809379; doi:10.1128/msphere.00883-21)

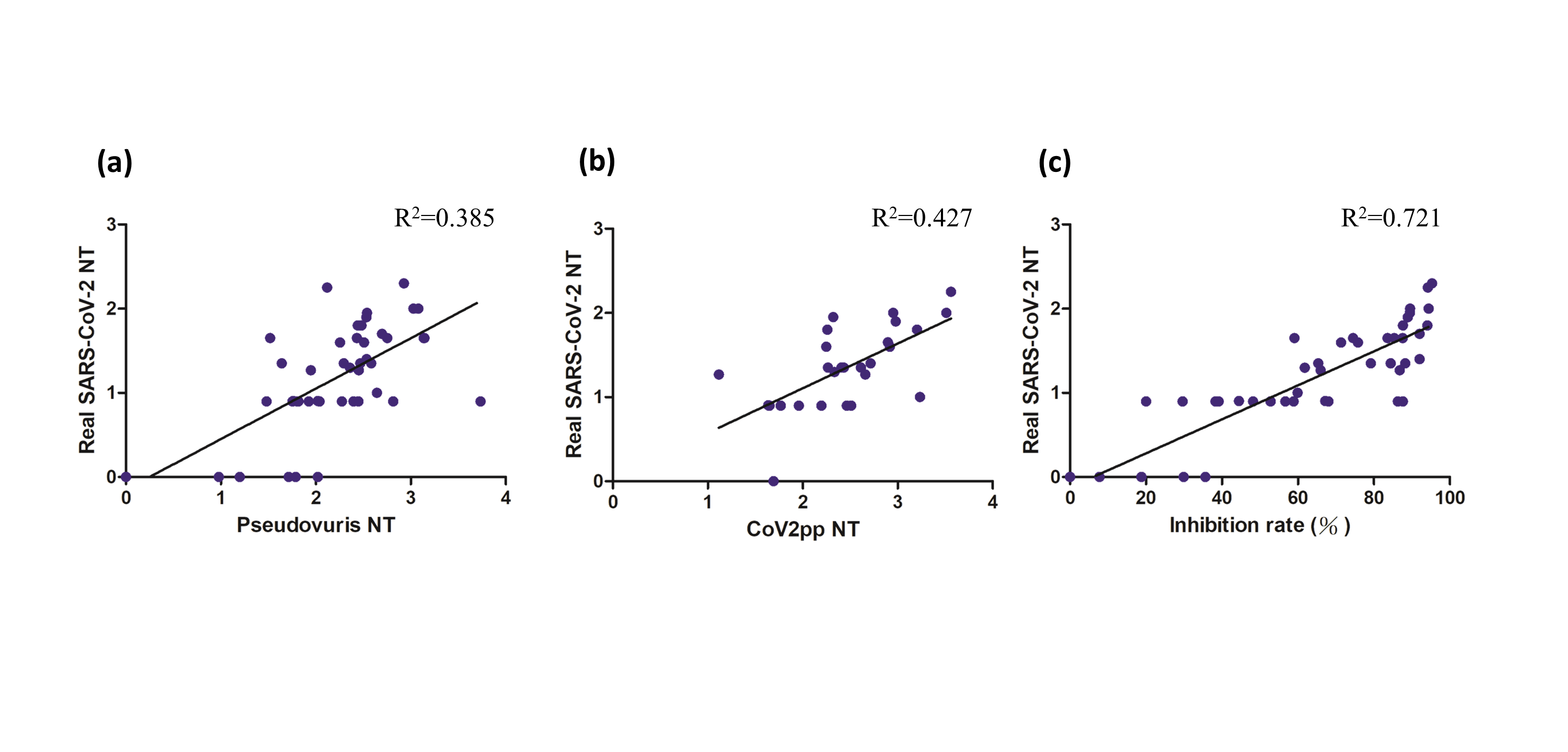

Supplement: FIG S1 [file msphere.00883-21-sf001.tif]
